# Randomised Study to Assess the Efficacy and Safety of Once-Daily Etravirine-Based Regimen as a Switching Strategy in HIV-Infected Patients Receiving a Protease Inhibitor–Containing Regimen. Etraswitch Study

**DOI:** 10.1371/journal.pone.0084676

**Published:** 2014-02-04

**Authors:** Patricia Echeverría, Anna Bonjoch, Jordi Puig, José Moltó, Roger Paredes, Guillem Sirera, Arelly Ornelas, Nuria Pérez-Álvarez, Bonaventura Clotet, Eugènia Negredo

**Affiliations:** 1 Department of HIV, Lluita contra la Sida Foundation, Germans Trias i Pujol University Hospital, Autonomous University of Barcelona, Barcelona, Spain; 2 Department of Econometrics, Statistics and Spanish Economy; University of Barcelona, Barcelona, Spain; 3 Statistics and Operations Research Department, Universitat Politècnica de Catalunya, Barcelona, Spain; 4 IrsiCaixa AIDS Research Institute-HIVACAT, Barcelona, Spain; University of Ottawa, Canada

## Abstract

**Background:**

Etravirine (ETR) was approved for patients with virological failure and antiretroviral resistance mutations. It has also shown antiviral efficacy in antiretroviral-naïve patients. However, data on the switching from protease inhibitors (PI) to ETR are lacking.

**Methods:**

HIV-1-infected patients with suppressed viral load (VL) during a PI-containing regimen (>12 months) and no previous virological failure were randomized to switch from the PI to ETR (400 mg/day, dissolved in water) (ETR group, n = 22) or to continue with the same regimen (control group, n = 21). Percentage of patients with VL≤50 copies/mL were assessed at week 48, as well as changes in CD4 T-cell counts and metabolic profile.

**Results:**

We included 43 patients [72.9% male, 46.3 (42.2; 50.6) years]. Two patients receiving ETR (grade-1 diarrhea and voluntary discontinuation) and another in the control group (simplification) discontinued therapy early. No patients presented virological failure (two consecutive VL>50 copies/mL); treatment was successful in 95.2% of the control group and 90.9% of the ETR group (intention-to-treat analysis, missing = failure) (p = 0.58). CD4+ T-cell counts did not significantly vary [+49 cells/µL in the ETR group (p = 0.25) and −4 cells/µL in the control group (p = 0.71)]. The ETR group showed significant reductions in cholesterol (p<0.001), triglycerides (p = <0.001), and glycemia (p = 0.03) and higher satisfaction (0–10 scale) (p = 0.04). Trough plasma concentrations of ETR were similar to observed in studies using ETR twice daily.

**Conclusion:**

Switch from a PI-based regimen to a once-daily combination based on ETR maintained undetectable VL during 48 weeks in virologically suppressed HIV-infected patients while lipid profile and patient satisfaction improved significantly.

**Trial Registration:**

ClinicalTrials.gov NCT01034917

## Introduction

Etravirine is a second-generation non-nucleoside analog reverse transcriptase inhibitor (NNRTI). It was approved by the U.S. Food and Drug Administration and the European Medicines Agency in 2008 for clinical use in adults with incomplete virologic suppression and resistance to multiple antiretroviral agents, including previous NNRTI, in combination with a ritonavir-boosted protease inhihitor (PI) [1]–[3].

The clinical recommendation for market authorization of etravirine was mainly based on the results of 2 pivotal phase 3 studies, DUET-1 and DUET-2 [4], [5]. The etravirine-containing arm showed higher rates of viral suppression than the placebo arm, and the likelihood of virologic failure was correlated with the number of baseline NNRTI and nucleoside analog reverse transcriptase inhibitor (NRTI) resistance mutations [4]–[9]. In addition, patients taking etravirine, administered twice daily, tolerated the regimen well, and the magnitude of the adverse events observed during treatment suggests that etravirine has a favorable safety and tolerability profile [4], [5], [10].

Since pharmacokinetic studies support the use of etravirine once daily [11]–[14], it appears to be a suitable option in antiretroviral simplification strategies. However, data on simplification with etravirine are lacking, and only 2 recently published studies have assessed switching from the NNRTI efavirenz to etravirine in virologically suppressed patients [15], [16]. Both studies showed the etravirine-containing regimen to be well tolerated and reported a significant improvement in efavirenz-related central nervous system symptoms. Nevertheless, we do not know whether patients with sustained undetectable HIV-1 RNA levels receiving a PI-based regimen can safely switch their current PI to etravirine. As nowadays antiretroviral treatment should be continued indefinitely, different effective and safe options, other than PI-based combinations, are required, mainly for those subjects suffering PI- or ritonavir-related toxicities or reporting an uncomfortable dosin Schedule with the regimen (twice daily dose, use of ritonavir…).

We designed a pilot study to test the efficacy and safety of switching from a PI to etravirine as an antiretroviral switching strategy in patients with a suppressed viral load. Our approach was based on the high antiviral potency and high genetic barrier to resistance of etravirine, as well as the favorable safety profile and patient-friendly dosing (once daily dose and dissolved in water).

## Methods

### Ethics Statement

The protocol for this trial and supporting CONSORT checklist are available as supporting information; see Checklist S1 and Protocol S1. The study protocol was approved by the Ethics Committee of our center “Germans Trias I Pujol Universitary Hospital. Barcelona, Spain” and local health authorities (Dr J. Costa, President of Clinical Research Ethics Committee), before the trial began and was undertaken in accordance with the Declaration of Helsinki and the requirements of Good Clinical Practice. (ClinicalTrials.gov ISRCTN 01034917). Study Protocol code: ETRA-SWITCH, N° 2009-016455-21 Eudra CT, version 1 (September, 29/2009).

Written informed consent was obtained from all participants.

### Study Design and Participants

We performed a 48-week, prospective, randomized, pilot study in HIV-1-infected outpatients. The study candidates were all those patients receiving standard triple-drug highly active antiretroviral therapy (HAART) including a PI for more than 12 months, with an undetectable viral load (VL <50 copies/mL) for more than 6 months and no documented resistance to NNRTIs or NRTIs or virological failure with previous regimens. For therapy to be switched to etravirine, patients had to fulfill at least 1 of the following criteria: dyslipidemia (low-density lipoprotein [LDL] cholesterol >130 mg/dL or triglycerides >150 mg/dL, according our reference laboratory, or current use of lipid-lowering agents), antiretroviral-related gastrointestinal disturbances, or a repeatedly expressed dissatisfaction with the current antiretroviral regimen.

### Randomization, Follow-up, and Assessment

Participants were randomly assigned in a ratio of 1∶1 to switch from the PI to etravirine (400 mg/day, dissolved in water; ETR group) or to continue with the same PI-based regimen (control group). The treatment allocation list was generated by blocks using numbers drawn from the uniform distribution.

Patients were assessed at week 0 (baseline) and weeks 4, 12, 24, 36 and 48. The laboratory data recorded at these time points included plasma HIV-1 RNA, CD4 and CD8 T-cell counts, biochemistry (fasting conditions) including a complete lipid profile (total cholesterol, LDL cholesterol, high-density lipoprotein cholesterol [HDL], triglycerides).

Virological failure was defined as the viral rebound (confirmed viral load >50 copies/mL). HIV-RNA was determined using the AMPLICOR HIV-1 MONITOR Test, v 1.5 (Roche Diagnostic, Basel, Switzerland).

HIV-related data (time since HIV diagnosis, risk behavior, nadir CD4 T-cell count, time on antiretroviral therapy, and time on NNRTIs, NRTIs, and PIs) were recorded from the patient’s records.

Adverse events and reasons for discontinuation were also recorded at the same time points. Adverse events were classified according to the definitions of the World Health Organization. Reasons for discontinuation were classified as follows: virological failure, adverse events (grade 1–2 or 3–4), death, or any other cause (eg, voluntary discontinuation and simplification).

Patient satisfaction with therapy was assessed using two 0–10 Likert scales (satisfaction with treatment and preference for treatment) at baseline and weeks 24 and 48.

Etravirine trough concentrations in plasma were also determined at week 12 in all patients who were receiving the drug.

### Study Objectives and End Points

The principal efficacy analyses (proportion of patients who maintained HIV-1 RNA ≤50 copies/ml at week 48) were performed on-treatment and intention-to-treat (missing equal to failure). Additionally, variations in CD4 and CD8 T-cell count at each time point from baseline were also compared between groups, as well as changes in metabolic parameters (lipid metabolism and glucose) and liver enzimes. Changes in lipid parameters after switching to etravirine were also evaluated according to the PI used at baseline. Safety was assessed by comparing the percentage of patients who discontinued the study during the 48 weeks of follow-up and the percentage of patients with serious adverse events (grade 3–4).

Patient satisfaction with treatment at weeks 24 and 48 was compared between treatment arms and also by stratifying participants according to the reason for switching at baseline.

Etravirine trough concentrations in plasma were described and compared with historical data from studies including subjects taking etravirine not dissolved in water.

### Statistical Analyses

The analysis of the study included comparisons between baseline and weeks 4, 12, 36 and 48 in each group and comparisons between groups (control versus ETR group) at each visit point.

Quantitative variables were expressed as the mean and standard deviation (SD) or as the median and interquartile range (IQR); qualitative variables were expressed as frequencies and percentages. Normally distributed continuous variables were compared using the *t* test; non-normally distributed variables were compared using the Mann-Whitney test. The dependent *t* test for paired samples or the Wilcoxon signed rank test was performed to assess the significance of changes observed over time. The chi-square test or Fisher exact test was used as appropriate to compare discrete variables.

Statistical analysis was performed with the SPSS 15.0 program (SPSS, Inc., Chicago Illinois, USA).

## Results

The study sample comprised 43 patients: 22 in the ETR group and 21 in the control group.

Epidemiological, clinical, and HIV-related characteristics were summarized in table 1. Overall, the median (IQR) age was 46.3 (42.2; 50.6) years and 72.9% of patients were male. The median time (IQR) since diagnosis of HIV infection was 12 (5.8; 16.7) years; the median time (IQR) on therapy and on PIs was 6.1 (3.5; 11.6) years and 4.9 (2.8; 8.18) years, respectively.

**Table 1:** Baseline epidemiological and HIV-related characteristics.

	ETR group (n = 22)	Control group (n = 21)
Age, years	47.4 (42; 51)	46.2 (41.8; 50)
Gender, male (%)	63.6	76.2
Time since HIV diagnosis, years	12.9 (5.9; 17.3)	8.1 (4.2; 16.5)
Time on antiretroviral therapy, years	8.3 (3.9; 12.1)	8.1 (4.2; 16.5)
Time on PI, years	3.6 (0.7; 8.2)	5.2 (3.4; 9.6)
CD4 cell count/µL	729 (512; 858)	711 (484; 931)
**Reason for change, n (%)**		
Dyslipemia	12 (54.5)	9 (42.9))
Gastrointestinal disturbances	3 (13.6)	2 (9.5)
Posology (BID)/RTV	7 (31.8)	10 (47.6)
Use of lipid-lowering agents (LLD) n%	4 (18.2)	2 (9.5)
**NNRTI, n (%)**		
Abacavir+Lamivudine	8 (36.4)	7 (33.3)
Tenofovir+Emtricitabine	13 (59)	13 (61.9)
Others combinations	1 (4.6)	1 (4.8)
**Baseline PI, n (%)**		
Atazanavir/RTV (n = 23)	11 (50.5)	12 (57.1)
Lopinavir/RTV (n = 11)	7 (31.8)	4 (19.0)
Fosamprenavir/RTV (n = 6)	3 (13.6)	3 (14.3)
Saquinavir/RTV (n = 3)	1 (4.5)	3 (9.5)
Darunavir/RTV (n = 0)	0	0
**Hepatitis C/B virus, n (%)**	3 (13.6)	4 (19)

Parameters are expressed as Median value (IQR 25;75) except when it was specified. HCV, hepatitis C virus; HBV, hepatitis B virus; PI, protease inhibitors; NNRTI, non-nucleoside analog reverse transcriptase inhibitor; RTV, ritonavir; BID, twice daily dosin.

The main reasons for participating in the study were dyslipidemia (48.8%), followed by low satisfaction with the previous regimen (twice-daily dosing and need to store ritonavir in a refrigerator) (39.5%) and gastrointestinal disorders (11.6%) (Table 1). At baseline, most patients were taking atazanavir and lopinavir/ritonavir based-regimen; the other PIs were fosamprenavir and saquinavir (Table 1).

### Virological and Immunological Outcome

The percentage of patients who maintained HIV-1 RNA ≤50 copies/ml at week 48 was similar between the groups, with no significant differences; 100% of patients who completed the study maintained HIV-RNA ≤50 copies/mL at week 48 in both groups, respectively (p = 0.84) (on-treatment analysis). The proportion in the intention-to-treat analysis (missing = failure) was 95.2% in the control group and 90.9% in the ETR group (p = 0.96). Difference of proportions between groups with 95% CI was 4.33(−10.75; 19.40).

Mean (SD) CD4 T-cell counts in the ETR group increased from 702 (333) to 749 (345) cells/µL, although the difference did not reach statistical significance (p = 0.25), and did not vary in the control group [from 717 (232) to 713 (270) cells/µL; p = 0.81]. No statistically significant differences were recorded between groups at any time point (p = 0.71 at week 48).

### Metabolic Changes

Patients from the ETR group showed significant reductions in cholesterol (from 207 to 191 mg/dL, p<0.001), triglycerides (from 186 to 132 mg/dL, p<0.001), and glycemia (from 97 to 93 mg/dL, p = 0.03) (Table 2). Differences between groups were recorded for triglycerides (p = 0.04) and glycemia (p = 0.03) at week 24. At week 48, no patients from the ETR group presented grade 3–4 elevations in total and LDL cholesterol while were recorded in 10.5% and 5.6%, respectively, of the control group; no differences were seen between groups in any of both parameters (p = 0.23 and p = 0.47, respectively). No patients showed grade 3–4 elevations in triglyceride levels at week 48.

**Table 2:** Changes in lipid profile and glycemia and satisfaction.

	ETR group			P value*	Control group			P value*
	Baseline (n = 22)	Week 24(n = 20 )	Week 48(n = 20)		Baseline(n = 21)	Week 24(n = 20 )	Week 48(n = 20)	
**Total cholesterol (mg/dL)**	207.2 (42.8)	186 (41)	190.8 (39.8)	**<0.001**	197.8 (47.4)	204.4 (42.9	199 (49.6)	0.71
**HDL-cholesterol (mg/dL)**	52.9 (18.3)	48 (14.5)	52.8 (20.4)	0.88	54.2 (19.6)	52.3 (21)	55.1 (24.9)	0.59
**CT/HDL-cholesterol ratio**	3.96	3.85	3.57	0.12	3.86	4.19	3.78	0.82
**LDL-cholesterol (mg/dL)**	116.6 (38.9)	113 (33)	110.5 (32.2)	0.10	114.6 (43.8)	121 (38.3)	113.6 (35.4)	0.50
**Triglycerides (mg/dL)**	186.4 (95.8)	119.4 (56)	132.4 (79.3)	**<0.001**	145.7 (106.4)	157 (9)	116.1 (58.5)	0.14
**Glycemia (mg/dL)**	97.4 (11.3)	94 (15)	92.7 (11.7)	**0.03**	90.3 (10.4)	88 (7.4)	88.0 (8.3)	0.22
**Satisfaction** **	7.7 (1.4)	8.9 (1.3)	9.2 (1.4)	**<0.01**	6 (2.3)	5.6 (2.4)	5.3 (2.1)	0.21

All values are shown as mean (SD).

* P values express intra-group differences between baseline and week 48.

Between groups, the only differences recorded were for triglycerides (p = 0.04) and glycemia (p = 0.03) at week 24.

HDL, High density lipoprotein; LDL, Low density lipoprotein.

** Patient satisfaction was assessed using two 0–10 likert scales.

When patients were stratified according to the PI used at baseline, a significant improvement was observed at week 48 in total cholesterol, triglycerides, and glycemia in patients who switched from lopinavir/ritonavir to etravirine (p = 0.01, p = 0.03, and p<0.0001, respectively). A significant difference was also observed in triglycerides in patients who switched from atazanavir (p = 0.04) and in glycemia in those who switched from fosamprenavir (p = 0.04).

When patients were stratified according to the presence of dyslipidemia or not at baseline, no difference were seen in lipid parameters (total cholesterol, p = 0.48; HDL-cholesterol, p = 0.44; LDL-cholesterol, p = 0.54; triglycerides, p = 0.54).

### Safety and Satisfaction

Therapy was discontinued early by 2 patients receiving etravirine (1 case of diarrhea (grade 1) and 1 voluntary discontinuation) and in 1 patient in the control group (change of regimen to simplify). No serious clinical adverse events (grade 3–4) were reported during the study.

No significant changes in liver enzymes were observed in either group, and no grade 3–4 elevations were recorded. No differences were seen between the groups at week 48 (aspartate aminotransferase, p = 0.77; alanine aminotransferase, p = 0.13).

Mean (SD) patient satisfaction rose from 7.7 (1.4) at baseline to 8.9 (1.3) at week 24 and 9.2 (1.4) at week 48 in the ETR group; these values decreased slightly in control patients from 6.0 (2.3) to 5.6 (2.4) and 5.3 (2.1), respectively (Table 2). Differences between groups were seen at weeks 24 (p = 0.001) and 48 (p = 0.04). These differences were marked when the subgroup of patients who switched regimen because of gastrointestinal disorders was analyzed: those who changed to etravirine reported higher satisfaction at weeks 24 and 48 than those who did not (p = 0.01 and 0.01, respectively).

The mean (range) etravirine trough concentrations in plasma was 569.3 (202–1141) ng/mL. Interpatient variability (% coefficient of variation) in etravirine concentrations was 50.5%.

## Discussion

Etravirine-based regimens could prove to be an excellent switching strategy in virologically suppressed HIV-infected patients thanks to their safety profile and patient-friendly dosing. Nonetheless, data on etravirine in this scenario are scarce.

At the end of the 1990s, many studies compared the antiviral outcome of an NNRTI (nevirapine or efavirenz) with the PIs used at the time [17]–[22]. Briefly, NNRTIs were non-inferior (in terms of antiviral potency) to PIs used in simplification strategies. Additionally, the lipid profile improved in most patients, especially with nevirapine [17]–[22]. Etravirine is a commercialized NNRTI that was approved to treat patients with incomplete virological suppression and resistance to previous NNRTI [4], [5]. Published evidence also shows that etravirine works well as first-line HIV therapy, as part of a once daily regimen, compared with efavirenz [23], [24]. This new-generation NNRTI induces few neuropsychiatric side effects and has a friendly lipid profile [23]–[25].

Nonetheless, despite its good safety profile, no experience has been reported with etravirine as an alternative therapy for simplification in virologically suppressed HIV-infected patients taking a ritonavir-boosted PI. The main reasons for switching from PIs in the clinical practice include PI-related laboratory toxicity (mainly metabolic abnormalities), clinical disorders (mainly gastrointestinal), and dosing-related conditions. In our study, the main reasons for switching were dyslipemia (almost half of all patients) and low satisfaction with the previous regimen (twice-daily dosing and the need to store ritonavir in a refrigerator) (around 40%). The low rate of patients who switched because of gastrointestinal disorders (11.6%) indicates that current regimens are generally well tolerated. The most frequently switched PI was atazanavir/ritonavir (56%), since, at the initiation of the study, ritonavir had to be kept in a refrigerator (a complaint from many patients).

Although this is a pilot study, our results revel that the switch from a PI to etravirine maintains virological suppression after 48 weeks of follow-up. No patients experienced viral rebound in either treatment arm. Moreover, CD4 T-cell counts increased slightly in patients who switched to etravirine. Although this increase was not statistically significant, it could be clinically relevant in some patients, especially in those with low CD4 T-cell counts.

As expected, and consistent with previous data for the effect of etravirine on lipid profile [4], [5], [23]–[25], the switch from a PI to etravirine led to an improvement in metabolic parameters. After 48 weeks, a significantly more favorable metabolic profile (decreased levels of total cholesterol, triglycerides, glycemia and no patients with grade 3–4 dyslipidemia at week 48) was observed in patients who switched to etravirine than in those who continued with a ritonavir-boosted PI. This improvement in metabolic parameters, including glycemia, was specially observed in those patients who switch to etravirine because of dyslipidemia, and especially marked in those patients who switched from lopinavir/ritonavir. Therefore, a favorable lipid profile is likely to reflect a class-wide association [26]–[32]. However, no improvement in HDL-cholesterol levels was observed in our participants after switching, a finding that is consistent with those of other studies on etravirine [25], but contrary to those observed with the other 2 NNRTIs, efavirenz and, mainly, nevirapine [26]–[32].

As for safety, the low rates of early withdrawal in the ETR arm that were similar to those in the control group support previous data on the good tolerability and low toxicity of etravirine [4], [5], [15], [16], [23], [24]; in fact, treatment failed in an even lower percentage of patients in our study than in others [15]. Only 1 patient interrupted the study prematurely because of toxicity (grade 1 diarrhea), which eventually did not seem to be associated with etravirine. A further 2 patients (1 in each arm) interrupted the study for other reasons (simplification and voluntary discontinuation). No grade 3–4 drug-related adverse events were reported in any patient receiving etravirine. As well, no significant increases in liver enzymes were recorded with the use of etravirine, even in patients with hepatitis C or B coinfection.

As patient satisfaction is generally high with current antiretroviral combinations, it is usually difficult to demonstrate differences after a switch. Among our patients, however, satisfaction improved significantly after switching to etravirine (even though the subgroup of patients evaluated was small), specifically in those with gastrointestinal disturbances at baseline. Nonetheless, some patients did not consider 4 tablets of etravirina (used in the study) plus the rest of the nucleoside backbone a simplified regimen. Marketing of 200-mg etravirine tablets has recently been approved in North America and in Europe. This new formulation will facilitate dosing and, consequently, patient acceptance and satisfaction. In addition, plasma concentrations of etravirine, administered once daily dissolved in water, were in the same range to those observed in subjects receiving undispersed etravirine tablets [11]–[14]. As well, all patients showed etravirine concentrations above the protein-binding adjusted 50% effective concentration 4 ng/mL. This data support the safety use of once daily dosing for etravirine, and the possibility of dispersing the tablets in order to enhance treatment convenience.

The limited sample size of the study does not permit to strongly state our conclusions but this pilot study provides initial data about the use of an etravirine-based regimen as a possible strategy in subjects with PI-associated toxicities or dissatisfied with their regimens. Additionally, this exploratory study allows us to establish guidelines for future studies with more patients. The randomized and controlled study designs, however, are both strengths of the study that becomes our results in useful information.

In conclusion, this randomized trial seems to demonstrate the maintenance of antiviral efficacy 48 weeks after switching from a PI to etravirine. Our results also confirm the favorable safety profile of etravirine, which has a lipid-friendly profile that is similar to that of the other commercialized NNRTIs and better than that of PI-containing regimens. Consequently, etravirine could be considered as an alternative in virologically suppressed HIV-infected patients receiving a PI-based antiretroviral combination and who have not experienced failure with other NNRTI-based regimens, mainly in those with metabolic disturbances.

## Supporting Information

Protocol S1 (DOC)

Checklist S1 (DOC)

## References

[1] Thompson M, Aberg J, Hoy J, Telenti A, Benson C, et al (2012) Antiretroviral treatment of adult HIV infection. 2012 recommendations of the International AIDS Society-USA panel. JAMA 308: 387–402. 22820792 10.1001/jama.2012.7961

[2] US Department for Health and Social Security (2011) Guidelines for the use of antiretroviral agents in HIV-1 infected adults and adolescents, January 10th 2011.http://www.aidsinfo.nih.gov/Guidelines/GuidelineDetail.aspx?GuidelineID=7. Accessed March 2011.

[3] European AIDS Clinical Society (EACS) (2011) Guidelines for the clinical management of HIV infected adults in Europe. http://www.europeanaidsclinicalsociety.org/guidelinespdf/1_Treatment_of_HIV_Infected_Adults.pdf Last accessed: May 2011.

[4] Madruga JV, Cahn P, Grinsztejn B, Haubrich R, Lalezari J, et al (2007) Efficacy and safety of TMC125 (etravirine) in treatment-experienced HIV-1-infected patients in DUET-1: 24-week results from a randomised, double-blind, placebo-controlled trial. Lancet 370(9581): 29–38. 17617270 10.1016/S0140-6736(07)61047-2

[5] Lazzarin A, Campbell T, Clotet B, Johnson M, Katlama C, et al (2007) Efficacy and safety of TMC125 (etravirine) in treatment-experienced HIV-1-infected patients in DUET-2: 24-week results from a randomised, double-blind, placebo-controlled trial. Lancet 370(9581): 39–48. 17617271 10.1016/S0140-6736(07)61048-4

[6] Martinez E, Nelson M (2010) Simplification of antiretroviral therapy with etravirine. AIDS Rev 12 (1): 52–9. 20216910

[7] Ruxrungtham K, Pedro RJ, Latiff GH, Conradie F, Domingo P, et al (2008) Impact of reverse transcriptase resistance on the efficacy of TMC125 (etravirine) with two nucleoside reverse transcriptase inhibitors in protease inhibitor-naïve, nonnucleoside reverse transcriptase inhibitor-experienced patients: study TMC125-C227. HIV Med 9(10): 883–96. 18795960 10.1111/j.1468-1293.2008.00644.x

[8] Schöller-Gyüre M, Kakuda TN, Raoof A, De Smedt G, Hoetelmans RM (2009) Clinical pharmacokinetics and pharmacodynamics of etravirine. Clin Pharmacokinet 48 (9): 561–74. 10.2165/10895940-000000000-00000 19725591

[9] Andries K, Azijn H, Thielemans T, Ludovici D, Kukla M, et al (2004) TMC125, a novel next-generation nonnucleoside reverse transcriptase inhibitor active against nonnucleoside reverse transcriptase inhibitor-resistant human immunodeficiency virus type 1. Antimicrob Agents Chemother 48(12): 4680–6. 15561844 10.1128/AAC.48.12.4680-4686.2004 PMC529207

[10] Katlama C, Haubrich R, Lalezari R, Lazzarin A, Madruga J, et al (2009) Efficacy and safety of etravirine in treatment- experienced, HIV-1 patients: pooled 48 week analysis of two randomized, controlled trials. AIDS 23: 2289–2300. 19710593 10.1097/QAD.0b013e3283316a5e

[11] Moltó J, Valle M, Miranda C, Cedeño S, Negredo E, et al (2012) Herb-Drug Interaction between Echinacea purpurea and Etravirine in HIV-Infected Patients. Antimicrob Agents Chemother 56 (10): 5328–31. 10.1128/AAC.01205-12 PMC3457385 22869560

[12] De Jesus E, Lalezari JP, Osiyemi OO, Ruane PJ, Ryan R, et al (2010) Pharmacokinetics of once-daily etravirine without and with once daily darunavir/ritonavir in antiretroviral-naive HIV type-1-infected adults. Antivir Ther 15 (5): 711–20. 10.3851/IMP1562 20710052

[13] Gruzdev B, Rakhmanova A, Doubovskaya E, Yakovlev A, Peeters M, et al (2003) A randomized, double-blind, placebo-controlled trial of TMC125 as 7-day monotherapy in antiretroviral naive, HIV-1 infected subjects. AIDS 17: 2487–94. 14600520 10.1097/00002030-200311210-00011

[14] Boffito M, Jackson A, Lamorde M, Back D, Watson V, et al (2009) Pharmacokinetics and safety of etravirine administered once or twice daily after 2 weeks treatment with efavirenz in healthy volunteers. J Acquir Immune Defic Syndr 52 (2): 222–7. 10.1097/QAI.0b013e3181b061d0 19620877

[15] Waters L, Fisher M, Winston A, Higgs C, Hadley W, et al (2011) A phase IV, double-blind, multicentre, randomized, placebo-controlled, pilot study to assess the feasibility of switching individuals receiving efavirenz with continuing central nervous system adverse events to etravirine. AIDS 25: 65–71. 21099666 10.1097/QAD.0b013e328341685b

[16] Nguyen A, Calmy A, Delhumeau C, Mercier IK, Cavassini M, et al (2011) A randomized cross-over study to compare efavirenz and etravirine treatment. AIDS 25: 57–63. 21076278 10.1097/QAD.0b013e32833f9f63

[17] Martinez E, Arnaiz JA, Podzamczer D, Dalmau D, Ribera E, et al (2003) Substitution of nevirapine, efavirenz, or abacavir for protease inhibitors in patients with human immunodeficiency virus infection. New England Journal of Medicine 349 Suppl 111036–46. 12968087 10.1056/NEJMoa021589

[18] Becker S, Rachlis A, Gill J (2001) Successful substitution of protease inhibitors with efavirenz (EFV) in patients with undetectable viral loads – a prospective, randomized, multicenter, open-label study (DMP 049). In the programme and abstracts of the 8TH Conference on Retroviruses and Opportunistic infections. Chicago IL, February 2001 [abstract 20].

[19] Raffi F, Bonnet B, Ferre V, Esnault JL, Perré P, et al (2000) Substitution of a nonnucleoside reverse transcriptase inhibitor for a protease inhibitor in the treatment of patient with undetectable plasma human immunodeficiency virus type 1 RNA. Clin Infect Dis 31: 1274–8. 11073763 10.1086/317424

[20] Ruiz L, Negredo E, Domingo P, Paredes R, Francia E, et al (2001) Antiretroviral treatment simplification with nevirapine in protease inhibitor-experienced patients with HIV-associated lipodystrophy: 1-year prospective follow-up of a multicenter, randomized, controlled study. J Acquir Immune Defic Syndr 27 Suppl 3229–36. 11464141 10.1097/00126334-200107010-00003

[21] Negredo E, Cruz L, Paredes R, Ruiz L, Fumaz CR, et al (2002) Virological, immunological, and clinical impact of switching from protease inhibitors to nevirapine or to efavirenz in patients with human immunodeficiency virus infection and long-lasting viral suppression. Clin Infect Dis 34: 504–10. 11797178 10.1086/324629

[22] Barreiro P, Soriano V, Blanco F, Casimiro C, de la Cruz JJ, et al (2000) Risk and benefits of replacing protease inhibitors by nevirapine in HIV-infected subjects under long-term successful triple combination therapy. AIDS 14: 807–812. 10839588 10.1097/00002030-200005050-00006

[23] Nelson M, Stellbrink HJ, Podzamczer D, Banhegyi D, Gazzard B, et al (2011) A comparison of neuropsychiatric adverse events during 12 weeks of treatment with etravirine and efavirenz in a treatment-naive, HIV-1-infected population. AIDS 25(3): 335–40. 21150563 10.1097/QAD.0b013e3283416873

[24] Gazzard B, Duvivier C, Zagler C, Castagna A, Hill A, et al (2011) Phase 2 double-blind, randomized trial of etravirine versus efavirenz in treatment-naive patients: 48-week results. AIDS 25(18): 2249–58. 21881478 10.1097/QAD.0b013e32834c4c06

[25] Fätkenheuer G, Duvivier C, Rieger A, Durant J, Rey D, et al (2012) SENSE Study Team. Lipid profiles for etravirine versus efavirenz in treatment-naive patients in the randomized, double-blind SENSE trial. J Antimicrob Chemother 67(3): 685–90. 22210755 10.1093/jac/dkr533

[26] Tohyama J, Billheimer JT, Fuki IV, Rothblat GH, Rader DJ, et al (2009) Effects of nevirapine and efavirenz on HDL cholesterol levels and reverse cholesterol transport in mice. Atherosclerosis 204(2): 418–23. 18990393 10.1016/j.atherosclerosis.2008.09.016 PMC2755296

[27] Barragan P, Fisac C, Podzamczer D (2006) Switching strategies to improve lipid profile and morphologic changes. AIDS Rev 8(4): 191–203. 17219734

[28] Young J, Weber R, Rickenbach M, Furrer H, Bernasconi E, et al (2005) Lipid profiles for antiretroviral-naïve patients starting PI- and NNRTI-based therapy in the Swiss HIV cohort study. Antivir Ther 10(5): 585–91. 16152752

[29] Fisac C, Fumero E, Crespo M, Roson B, Ferrer E, et al (2005) Metabolic benefits 24 months after replacing a protease inhibitor with abacavir, efavirenz or nevirapine. AIDS 19(9): 917–25. 15905672 10.1097/01.aids.0000171405.46113.bf

[30] Van Leth F, Phanuphak P, Stroes E, Gazzard B, Cahn P, et al (2004) Nevirapine and efavirenz elicit different changes in lipid profiles in antiretroviral-therapy-naive patients infected with HIV-1. PloS Med 1(1): e19. 15526045 10.1371/journal.pmed.0010019 PMC523838

[31] Negredo E, Ribalta J, Ferré R, Salazar J, Rey-Joly C, et al (2004) Efavirenz induces a striking and generalized increase of HDL-cholesterol in HIV-infected patients. AIDS 18(5): 819–21. 15075521 10.1097/00002030-200403260-00017

[32] Negredo E, Ribalta J, Paredes R, Ferré R, Sirera G, et al (2002) Reversal of atherogenic lipoprotein profile in HIV-1 infected patients with lipodystrophy after replacing protease inhibitors by nevirapine. AIDS 16(10): 1383–9. 12131215 10.1097/00002030-200207050-00010

