# Supplementary material for: Randomised Study to Assess the Efficacy and Safety of Once-Daily Etravirine-Based Regimen as a Switching Strategy in HIV-Infected Patients Receiving a Protease Inhibitor–Containing Regimen. Etraswitch Study
Source: PLoS One. 2014 Feb 4;9(2):e84676. doi: 10.1371/journal.pone.0084676 (PMC3913576; doi:10.1371/journal.pone.0084676)
Supplement: Protocol S1 — (DOC) [file pone.0084676.s001.doc]

**PILOT STUDY TO ASSESS THE EFFICACY AND SAFETY OF SWITCHING PROTEASE INHIBITOR TO ETRAVIRINE IN HIV-1-INFECTED SUBJECTS WITH VIREMIA SUPPRESSION**

**Code: ETRA-SWITCH**

**EudraCT number: 2009-016455-21**

**Version 1, 23^th^ September 2009**

**Sponsor:** *Lluita contra la SIDA Foundation*

Germans Trias i Pujol University Hospital

Carretera de Canyet s/n

08916 Badalona (Barcelona)

**Principal investigator:** Dra. Eugènia Negredo

**Co-investigators in Spain:**  Dra. Patricia Echeverría

Dr. Bonaventura Clotet

The information contained in this document is confidential and must not be revealed to third persons without prior authorization as contemplated by Law.

**SIGNATURES**

The coordinating investigator, the sponsor and the monitor of the study:

PILOT STUDY TO ASSESS THE EFFICACY AND SAFETY OF SWITCHING PROTEASE INHIBITOR TO ETRAVIRINE IN HIV-1-INFECTED SUBJECTS WITH VIREMIA SUPPRESSION

Declare that this study will be conducted in compliance with the protocol, Good Clinical Practices (GCP) and the applicable regulatory requirements.

Principal Investigator:

Dra. Eugènia Negredo

Signature and Date:

Sponsor:

Dr. Bonaventura Clotet, Lluita contra la SIDA Foundation

Signature and Date:

# 1.- SUMMARY

## 1.0. TYPE OF APPLICATION

Clinical trial including a marketed investigational product under different conditions from the authorised.

## 1.1. SPONSOR IDENTIFICATION

*Lluita Contra la SIDA Foundation*

Carretera de Canyet, s/n

08916 – Badalona (Barcelona)

Telephone: +34 93 497 88 87

Fax: +34 93 465 76 02

[www.flsida.org](http://www.flsida.org/)

## 1.2. TITLE OF THE CLINICAL TRIAL

PILOT STUDY TO ASSESS THE EFFICACY AND SAFETY OF SWITCHING PROTEASE INHIBITOR TO ETRAVIRINE IN HIV-1-INFECTED SUBJECTS WITH VIREMIA SUPPRESSION

## 1.3. PROTOCOL CODE

ETRA-SWITCH

## 1.4. PRINCIPAL INVESTIGATOR

Dra. Eugènia Negredo

Lluita contra la Sida Foundation, HIV Unit

Hospital Universitari Germans Trias i Pujol

Ctra. de Canyet, s/n

Badalona, Barcelona, Spain

[enegredo@flsida.org](mailto:enegredo@flsida.org)

T. +34 93 497 88 87

F. +34 93 465 76 02

## 1.5. STUDY CENTERS

Hospital Universitari Germans Trias i Pujol. Badalona, Spain

## 1.6. ETHICS COMMITTEE APPROVING THE TRIAL

CEIC Hospital Universitari Germans Trias i Pujol.

## 1.7. MONITOR

Roser Escrig / Sílvia Gel

FLS-Research Support

Hospital Universitari Germans Trias i Pujol

Ctra. de Canyet, s/n

Badalona, Barcelona, Spain

## 1.8. EXPERIMENTAL DRUG

Intelence ® - Etravirine 100mg tablets (400 mg dissolved in water every 24 h)

## 1.10. CLINICAL TRIAL PHASE

Phase III, pilot study.

## 1.11. OBJECTIVE

To compare the virological efficacy of etravirine-based regimen with standard PI-containing regimens.

## 1.12. STUDY DESIGN

A 48 week randomized, active controlled, open-label, parallel, proof-of-concept pilot clinical trial.

## 1.13. STUDY PATHOLOGY

Patients with HIV-1 infection.

## 1.14. PRINCIPAL ASSESSMENT VARIABLE

Viral load.

## 1.15. TYPE OF POPULATION AND NUMBER OF PATIENTS

HIV-infected patients on HAART regimen including a protease inhibitor and at least two nucleos/tide inhibitors.

A total sample size of 40 patients: 20 in Control group and 20 in Etravirine group.

## 1.16. DURATION OF TREATMENT

48 weeks.

## 1.17. CALENDAR AND CONCLUSION DATE

The foreseen date of the inclusion of the first patient is January 2010 and the conclusion of treatment of the last patient will be, approximately, June 2011. The final report will be presented on June 2012.

# 2.- TABLE OF CONTENTS

[1.- SUMMARY 3](#__RefHeading___Toc242081683)

[2.- TABLE OF CONTENTS 5](#__RefHeading___Toc242081701)

[3.- GENERAL INFORMATION 7](#__RefHeading___Toc242081702)

[3.1. TRIAL IDENTIFICATION 7](#__RefHeading___Toc242081703)

[3.2. TYPE OF TRIAL 7](#__RefHeading___Toc242081704)

[3.3. DESCRIPTION OF THE STUDY PRODUCTS 7](#__RefHeading___Toc242081705)

[3.4. SPONSOR INFORMATION 7](#__RefHeading___Toc242081706)

[3.5. TECHNICAL DIRECTOR SUPERVISING SAMPLE PREPARATION AND CONTROL 7](#__RefHeading___Toc242081707)

[3.6. MONITOR IDENTIFICATION 7](#__RefHeading___Toc242081708)

[3.7. CENTERS WHERE THE TRIAL WILL BE CONDUCTED AND INFORMATION ON THE INVESTIGATORS PARTICIPATING IN THE TRIAL 7](#__RefHeading___Toc242081709)

[3.8. TECHNICAL SERVICES 7](#__RefHeading___Toc242081710)

[3.9. EXPECTED TRIAL DURATION 7](#__RefHeading___Toc242081711)

[4.- RATIONALE AND AIMS 8](#__RefHeading___Toc242081712)

[4.1. RATIONALE 8](#__RefHeading___Toc242081713)

[4.2. AIMS 9](#__RefHeading___Toc242081714)

[5.- TYPE OF CLINICAL TRIAL AND DESIGN 10](#__RefHeading___Toc242081715)

[5.1. CLINICAL TRIAL DEVELOPMENT PHASE 10](#__RefHeading___Toc242081716)

[5.2. CLINICAL TRIAL DESIGN 10](#__RefHeading___Toc242081717)

[5.3. RANDOMIZATION AND STRATIFICATION PROCESS 10](#__RefHeading___Toc242081718)

[5.4. STUDY DURATION 10](#__RefHeading___Toc242081719)

[5.6. STUDY PLAN 10](#__RefHeading___Toc242081720)

[5.7. MASKING TECHNIQUE 10](#__RefHeading___Toc242081721)

[5.8. CASE REPORT FORM 10](#__RefHeading___Toc242081722)

[6. – SELECTION OF PATIENTS 11](#__RefHeading___Toc242081723)

[6.1. CRITERIA FOR DIAGNOSING THE STUDY PATHOLOGY 11](#__RefHeading___Toc242081724)

[6.2. INCLUSION CRITERIA 11](#__RefHeading___Toc242081725)

[6.3. EXCLUSION CRITERIA 11](#__RefHeading___Toc242081726)

[6.4. NUMBER OF PATIENTS 11](#__RefHeading___Toc242081727)

[6.5. SECURITY CRITERIA 11](#__RefHeading___Toc242081728)

[6.6. WITHDRAWAL AND DROPOUT CRITERIA 12](#__RefHeading___Toc242081729)

[6.7. PRE-RANDOMIZATION LOSSES 12](#__RefHeading___Toc242081731)

[6.8. APPROXIMATE DURATION OF THE RECRUITMENT PERIOD 12](#__RefHeading___Toc242081732)

[7.- TREATMENT DESCRIPTION 13](#__RefHeading___Toc242081733)

[7.1. EXPERIMENTAL AND CONTROL TREATMENTS 13](#__RefHeading___Toc242081734)

[7.2. SUPPLY, PACKAGING, LABELING AND STORAGE 13](#__RefHeading___Toc242081735)

[7.3. DOSAGE, INTERVAL, ROUTE AND FORM OF ADMINISTRATION 13](#__RefHeading___Toc242081736)

[7.4. MODIFICATION OF THE TREATMENT REGIMEN 13](#__RefHeading___Toc242081737)

[7.5. CONCOMITANT TREATMENTS 13](#__RefHeading___Toc242081738)

[7.6. COMPLIANCE 14](#__RefHeading___Toc242081739)

[7.7. NORMS FOR MANIPULATING THE STUDY TREATMENTS 14](#__RefHeading___Toc242081740)

[8.- TRIAL CONDUCTION AND RESPONSE EVALUATION 15](#__RefHeading___Toc242081741)

[8.1. CRITERIA FOR RESPONSE EVALUATION 15](#__RefHeading___Toc242081742)

[8.2. PROCEDURES FOR THE STUDY PARAMETERS 16](#__RefHeading___Toc242081746)

[8.3. TRIAL DEVELOPMENT 17](#__RefHeading___Toc242081749)

[9.- ADVERSE EVENTS 18](#__RefHeading___Toc242081750)

[9.1. DEFINITION 18](#__RefHeading___Toc242081751)

[9.2. DESCRIPTION OF THE IMPUTABILITY CRITERIA 18](#__RefHeading___Toc242081752)

[9.3. PROCEDURE FOR REPORTING ADVERSE EVENTS 19](#__RefHeading___Toc242081753)

[10.- ETHICAL ASPECTS 21](#__RefHeading___Toc242081754)

[10.1. GENERAL CONSIDERATIONS 21](#__RefHeading___Toc242081755)

[10.2. PATIENT INFORMATION AND INFORMED CONSENT 21](#__RefHeading___Toc242081758)

[10.3. CONFIDENTIALITY AND PATIENT PROTECTION 21](#__RefHeading___Toc242081759)

[11.- PRACTICAL ASPECTS 22](#__RefHeading___Toc242081760)

[11.1. RESPONSABILITIES OF STUDY PARTICIPANTS 22](#__RefHeading___Toc242081761)

[11.2. PROTOCOL AMENDMENTS 22](#__RefHeading___Toc242081762)

[11.3. DATA COLLECTION AND FILING 22](#__RefHeading___Toc242081763)

[11.4. GOOD CLINICAL PRACTICE (GCP) 22](#__RefHeading___Toc242081764)

[11.5 PUBLICATION 22](#__RefHeading___Toc242081765)

[11.6. STUDY DOCUMENTATION 22](#__RefHeading___Toc242081766)

[12.- STATISTICAL ANALYSIS 23](#__RefHeading___Toc242081767)

[12.1. SAMPLE DESCRIPTION 23](#__RefHeading___Toc242081768)

[12.2. RESPONSE EVALUATION 23](#__RefHeading___Toc242081769)

[12.3. SAFETY ANALYSIS 23](#__RefHeading___Toc242081770)

[12.4. SAMPLE SIZE DESCRIPTION 23](#__RefHeading___Toc242081771)

[Appendix I: CASE REPORT FORM (CRF) 24](#__RefHeading___Toc242081772)

[Appendix II: INVESTIGATOR’S BROCHURE 25](#__RefHeading___Toc242081773)

[Appendix III: PATIENT INFORMATION AND WRITTEN INFORMED CONSENT 26](#__RefHeading___Toc242081774)

[Appendix IV: INSURANCE 27](#__RefHeading___Toc242081775)

[Appendix V: NOTIFICATION FORM OF SERIOUS ADVERSE EVENTS 28](#__RefHeading___Toc242081776)

[Appendix VI: REFERENCES 29](#__RefHeading___Toc242081777)

# 3.- GENERAL INFORMATION

## 3.1. TRIAL IDENTIFICATION

Code: ETRA-SWITCH

Title of the study: PILOT STUDY TO ASSESS THE EFFICACY AND SAFETY OF SWITCHING PROTEASE INHIBITOR TO ETRAVIRINE IN HIV-1-INFECTED SUBJECTS WITH VIREMIA SUPPRESSION

## 3.2. TYPE OF TRIAL

Clinical trial including a marketed investigational product under different conditions from the authorised.

## 3.3. DESCRIPTION OF THE STUDY PRODUCTS

Intelence ® - Etravirine 100mg tablets (400 mg dissolved in water every 24 h)

## 3.4. SPONSOR INFORMATION

*Lluita Contra la SIDA Foundation*

Ctra. de Canyet, s/n

08916, Badalona (Barcelona) SPAIN

Telephone: + 34 93 497 88 87

Fax: + 34 93 465 76 02

[www.flsida.org](http://www.flsida.org/)

## 3.5. TECHNICAL DIRECTOR SUPERVISING SAMPLE PREPARATION AND CONTROL

The technical director from the manufacturer lab (Janssen-Cilag SpA).

## 3.6. MONITOR IDENTIFICATION

Roser Escrig / Sílvia Gel

*FLS-Research Support*

Ctra. de Canyet, s/n

08916 – Badalona (Barcelona) SPAIN

Telephone: + 34 93 497 84 14 / +34 629 77 36 20

Fax: + 34 93 465 76 02

[rescrig@fls-rs.com](mailto:rescrig@fls-rs.com)

## 3.7. CENTERS WHERE THE TRIAL WILL BE CONDUCTED AND INFORMATION ON THE INVESTIGATORS PARTICIPATING IN THE TRIAL

The trial will be conducted in the Germans Trias i Pujol Hospital and the investigators participating on it will be Dr. Eugènia Negredo, Dr. Patricia Echeverría, Dr. Bonaventura Clotet and the nurse Jordi Puig.

## 3.8. TECHNICAL SERVICES

Biochemistry, Haematology, Viral Load and CD4 counts will be performed in the central laboratories of the participating Hospital.

Genotypic test will be done in the irsiCaixa Retrovirology Laboratory.

## 3.9. EXPECTED TRIAL DURATION

The maximum study duration will be 72 weeks: 24 weeks of inclusion and 48 weeks of follow-up.

# 4.- RATIONALE AND AIMS

## 4.1. RATIONALE

Etravirine is a second generation non-nucleoside analogue reverse transcriptase inhibitor (NNRTI) approved by the U.S. Food and Drug Administration (FDA) in January 2008 and by the European Medicines Agency in September 2008 for clinical use in adults with incomplete virologic suppression and resistance to previous NNRTI and other antiretroviral classes. ^1,2^

Etravirine has shown *in vitro* activity against viral strains with mutations that confer resistance to efavirenz and nevirapine.^2^ However, etravirine's activity is strongly affected by the number of NNRTI mutations present, as well as by the specific mutations. For this reason, in the clinical practice it is important to discontinue the NNRTI as early as possible in patients who have developed resistance to a first-generation NNRTI, in order to prevent the selection of additional resistance mutations that would diminish the efficacy of etravirine.^3^

The clinical recommendation for market authorization of etravirine was based on the results of two pivotal phase 3 studies, DUET-1 and DUET-2, and four supportive phase 2b trials. Both DUET studies were randomized, double-blind, placebo-controlled, international trials. These studies were designed to evaluate the long-term efficacy, tolerability, and safety of etravirine compared to placebo as part of optimized background regimen including darunavir/ritonavir (DRV/rtv) in treatment-experienced HIV-1 infected subjects with NNRTI resistance after failure of an initial NNRTI-based regimen. The arm including etravirine showed high rates of viral suppression and the likelihood of virologic failure in the etravirine group was correlated with the number of baseline NNRTI and nucleoside analogue reverse transcriptase inhibitor NRTI mutations.^4,5,6,7,8^

Based on these results, the use of etravirine should not be recommended with 2-NRTIs without additional active agents in patients with NNRTI resistance mutations.^3,4^

Additionally, etravirine is a substrate and induces of CYP 3A4, as well as being a substrate and inhibitor of 2C9, and 2C19, with a complex drug interaction potential. Pharmacokinetic studies show either etravirine or the co-administered antiretroviral may be significantly affected when used in combination. As a result, etravirine is not recommended to be used with any unboosted protease inhibitor (PI).

A question not yet explored is whether subjects with sustained undetectable HIV-1 RNA-levels experiencing antiretroviral-related toxicity can safely switch their current PI to etravirine. This treatment strategy could allow improvements in tolerability and lipid profile and would permit an easy posology. We designed a proof-of-concept study to test the efficacy and safety of switching from a PI to etravirine in subjects with viral suppression as an antiretroviral strategy of simplification therapy, based on the high antiviral potency, low toxicity, together with its easy posology (even in water dissolution).

***Hypothesis***

- Hypothesis 1. Switching from a PI to etravirine, as a simplification strategy, in subjects with maintained viral suppression will be associated with comparable rates of virological suppression.
- Hypothesis 2. Switching to etravirine in this clinical context will be associated with an improvement of lipid parameters and patient’s satisfaction in comparison with remaining on the previous PI-based regimen.

## 4.2. AIMS

Primary objective:

To compare the virological efficacy of etravirine-based regimen with standard PI-containing regimens.

Secondary objectives:

To compare the immunological efficacy of etravirine-based regimen with standard PI-containing regimens.

To compare the tolerability and toxicity of etravirine-based regimen with standard PI-containing regimens.

To compare the cardiovascular risk of etravirine-based regimen with standard PI-containing regimens.

To compare patient’s satisfaction of etravirine-based regimen with standard PI-containing regimens.

# 5.- TYPE OF CLINICAL TRIAL AND DESIGN

## 5.1. CLINICAL TRIAL DEVELOPMENT PHASE

Phase III, pilot study.

## 5.2. CLINICAL TRIAL DESIGN

A 48 week randomized, active controlled, open-label, parallel, proof-of-concept pilot clinical trial.

## 5.3. RANDOMIZATION AND STRATIFICATION PROCESS

Patients receiving a triple-drug standard HAART PI-based and will be randomly assigned in a 1:1 ratio:

- To switch from the PI to Etravirine (Etravirine group), or
- Continue with the same regimen (Control group).

No stratification process will be used.

## 5.4. STUDY DURATION

The total duration of the study will be 72 weeks: 24 weeks for patient inclusion and 48 weeks of follow-up once the last patient is included.

## 5.6. STUDY PLAN

Subjects fulfilling the inclusion criteria will be asked to participate in this study and if they agree, signed informed consent will be obtained. After accepting the participation, patients would be randomized and assessed the first visit. Study visits will be at weeks 4, 12, 24, 36 and 48.

## 5.7. MASKING TECHNIQUE

Not applicable, since this has an open clinical trial design.

## 5.8. CASE REPORT FORM

Content of the case report form (CRF) is attached in appendix I.

# 6. – SELECTION OF PATIENTS

## 6.1. CRITERIA FOR DIAGNOSING THE STUDY PATHOLOGY

The diagnostic criteria are based in the clinical history, physical examination, and laboratory parameters.

## 6.2. INCLUSION CRITERIA

1. Adult patient having a diagnosis of HIV-1 infection.
2. Antiretroviral therapy started at least 12 months before, always with a HAART combination including 2 NRTIs plus a PI
3. Current HAART combination including 2 NRTIs plus a PI.
4. Maintained undetectable plasma HIV-1 RNA (VL < 50 copies/mL) since the beginning of antiretroviral therapy, for at least 6 months.
5. Absence of suspected or documented resistance mutations in the RT associated to NNRTIs or to any NRTI.
6. Patient having at least one of the following conditions:

- Dyslipemia (LDL cholesterol >130 mg/dL or triglycerides > 350 mg/dL) derived from their current PI regimen or current use of lipid lowering agents due to dyslipemia,
- Antiretroviral-related gastrointestinal disturbances, or
- Low patient’s satisfaction associated with the current regimen posology (BID regimen, ritonavir use, ritonavir intolerance…).

1. Good treatment adherence.
2. Voluntary written informed consent.

## 6.3. EXCLUSION CRITERIA

1. Previous therapy with mono or dual antiretroviral therapies at least 6 months before.
2. Previous antiretroviral treatment failures, treatment interruptions or blips in viral load (VL > 50 copies/mL) at least 6 months before.
3. Acute infections or uncontrolled chronic infection in the 2 months previous to the inclusion.
4. Pregnancy or fertile women willing to be pregnant.
5. Clinically significant malabsorption syndrome within 30 days prior to randomization.

## 6.4. NUMBER OF PATIENTS

The study is going to be carried out in a sample 40 patients: 20 in Control group and 20 in Etravirine group.

## 6.5. SECURITY CRITERIA

The following safety procedures will be implemented:

- HIV-1 RNA levels will be performed in both treatment arms at week 4 of the study and every 12 weeks thereafter up to week 48.
- An interim evaluation will be performed when 75% of subjects reach week 24 of follow-up. The study will be prematurely interrupted at week 24 if the difference in rates of virological failure between study arms is 10% or higher.

## 6.6. WITHDRAWAL AND DROPOUT CRITERIA

The patients will complete the clinical study before the stipulated time in the following circumstances:

- Virologic failure: it is defined as an increase in VL > 50 copies/mL on two consecutive visits.
- Interruption of treatment due to adverse events, intolerance or poor adherence during the study.
- Concurrent process or illness which in the opinion of the investigator requires the withdrawal of the patient.
- The patient does not wish to continue in the study.

If the study must be interrupted prematurely, all of the materials should be returned to the sponsor, at the *Lluita Contra la SIDA Foundation.*

### 6.6.1. Medical Approach to Withdrawal

In all cases in which the treatment is concluded prematurely for any reason, detailed information will be given about the date and reasons of the discontinuation to the promoter.

The investigator will facilitate the necessary medical support to the patient who discontinues the study prematurely due to treatment failure or to the appearance of an adverse event or a related concurrent illness.

In case of virologic failure,

- Genotypic resistance testing will be performed to all participants experiencing virologic failure during the study.
- A salvage regimen will be optimized according to genotypic resistance testing and previous antiretroviral treatment history.

## 6.7. PRE-RANDOMIZATION LOSSES

Do not proceed, patients who do not fulfill the selection criteria, will not be asked to participate in the study.

## 6.8. APPROXIMATE DURATION OF THE RECRUITMENT PERIOD

24 weeks.

# 7.- TREATMENT DESCRIPTION

## 7.1. EXPERIMENTAL AND CONTROL TREATMENTS

Patients in the control group will be receiving a standard triple-drug HAART composed of marketed drugs that will include a PI plus at least two NRTIs.

Patients in the experimental group will be receiving etravirine plus at least two NRTIs.

The experimental drug is etravirine, administered in a 100 mg tablet dissolved in water, 4 tablets (400mg) every 24 hours. The tablet formulation also contains Hypromellose, Microcrystalline cellulose, Colloidal anhydrous sílica, Croscarmellose sodium, Magnesium stearate, Lactose monohydrate.

## 7.2. SUPPLY, PACKAGING, LABELING AND STORAGE

All antiretroviral treatments will be administered by the Pharmacy Service in the marketed format.

All of the medication for the clinical study should be stored in a secure place during the study.

## 7.3. DOSAGE, INTERVAL, ROUTE AND FORM OF ADMINISTRATION

All candidates will be receiving a triple-drug standard HAART with marketed drugs and at approved standard doses that will include a PI plus at least two NRTIs.

Experimental group: switch from the PI to etravirine*.

Control group: to continue with their same regimen.

*Etravirine: 4 tablets of 100mg each (400mg), dissolved in water, every 24h

All the drugs will be orally administered.

## 7.4. MODIFICATION OF THE TREATMENT REGIMEN

No changes in treatment guidelines are foreseen.

## 7.5. CONCOMITANT TREATMENTS

All other treatments apart from antiretroviral medication administered during the study period will be considered concomitant treatments and should be documented in the CRF.

It is remembered that the patients who participate in the study should not continue any concomitant treatment without the knowledge and permission of the investigator.

Following known interactions with medicinal products are listed:

| **Medicinal product** | **Clinical comment** |
| --- | --- |
| Digoxin | It is recommended that digoxin levels be monitored when digoxin is combined with etravirine. |
| Amiodarone  Bepridil  Disopyramide  Flecainide  Lidocaine (systemic)  Mexiletine  Propafenone  Quinidine | Caution is warranted and therapeutic concentration monitoring, if available, is recommended for antiarrhythmics when co-administered with etravirine. |
| Clarithromycin | Alternatives to clarithromycin should be considered for the treatment of *Mycobacterium avium* omplex. |
| Warfarin | It is recommended that the international normalised ratio (INR) be monitored when warfarin is combined with etravirine. |
| Rifampicin  Rifapentine | Combination not recommended. |
| Rifabutin | The combination of etravirine and rifabutin should be used with caution due to the risk of decrease in etravirine and rifabutin exposures. |
| Diazepam | Alternatives to diazepam should be considered. |
| Dexamethasone (systemic) | Systemic dexamethasone should be used with caution or alternatives should be considered, particularly for chronic use. |
| St John's wort *(Hypericum perforatum)* | Combination not recommended. |
| Atorvastatin | The combination of etravirine and atorvastatin  can be given without any dose adjustments, however, the dose of atorvastatin may need to be altered based on clinical response. |
| Fluvastatin  Lovastatin  Pravastatin  Rosuvastatin  Simvastatin | Dose adjustments for these HMG Co-A reductase inhibitors may be necessary. |
| Cyclosporine  Sirolimus  Tacrolimus | Co-administration with systemic immunosuppressants should be done with caution because plasma concentrations of cyclosporine, sirolimus or tacrolimus may be affected when co-administered with etravirine. |
| Sildenafil  Vardenafil  Tadalafil | Concomitant use of PDE-5 inhibitors with etravirine may require dose adjustment of the PDE-5 inhibitor to attain the desired clinical effect. |

## 7.6. COMPLIANCE

The investigator is to ask the patient about treatment adherence to antiretroviral and concomitant treatment (particularly lipid-lowering drug agents) and this data is to be written in the clinical record. This data will be to guarantee the compliance.

## 7.7. NORMS FOR MANIPULATING THE STUDY TREATMENTS

Etravirine:

Store in the original bottle. Keep the bottle tightly closed in order to protect from moisture. Do not remove the desiccant pouches.

# 8.- TRIAL CONDUCTION AND RESPONSE EVALUATION

## 8.1. CRITERIA FOR RESPONSE EVALUATION

### 8.1.1. Principal Study Variable

Viral load at week 48.

Virologic failure is defined as an increase in HIV RNA >50 copies/mL in 2 determinations within 1 month. The first date with VL > 50 copies/mL will be used to calculate time to virologic failure.

### 8.1.2. Secondary Study Variables

- CD4+/CD8+ T lymphocytes count.
- Genotypic test if virologic failure occurs.
- Lipid profile: total, HDL-, LDL-cholesterol and triglyceride levels
- Administration of lipid-lowering drugs throughout the study (new administrations or the withdrawal of previous lipid-lowering drugs).
- Cardiovascular risk assessed by the SCORE equation
- Patient’s satisfaction assessed using a 100 mm analogue visual scale.
- Adverse events related to antiretroviral treatment (Toxicity)

### 8.1.3. Endpoints

**Primary objective:**

To compare the virological efficacy of etravirine-based regimen with standard PI-containing regimens.

- The percentage of patients who experienced virologic failure throughout the 48 weeks of the study. Virologic failure will be defined as an increase in HIV RNA >50 copies in 2 determinations within 1 month.
- Time to virologic failure, defined as an increase in HIV RNA >50 copies in 2 determinations within 1 month. The first date with VL > 50 will be used to calculate time to virologic failure.
- Determination of antiretroviral resistance at the time of virological failure and comparison with baseline.

**Secondary objectives:**

To compare the immunological efficacy of etravirine-based regimen with standard PI-containing regimens.

- Changes in CD4+/CD8+ T lymphocytes at week 48 from baseline.

To compare the tolerability and toxicity of etravirine-based regimen with standard PI-containing regimens.

- Changes in lipid parameters (total, HDL-, LDL-cholesterol and triglyceride levels) at week 48 relative to baseline values.
- Changes in the administration of lipid-lowering drugs throughout the study (new administrations or the withdrawal of previous lipid-lowering drugs).
- Percentage of patients who withdraw from the study.
- Percentage of patients who withdraw from the study due to toxicity.
- Percentage of patients with toxicity ≥ grade 3.

To compare the cardiovascular risk of etravirine-based regimen with standard PI-containing regimens.

- Changes in cardiovascular risk estimated by changes in the SCORE equation at week 48 relative to baseline values.

To compare patient’s satisfaction of etravirine-based regimen with standard PI-containing regimens.

- Changes in patient’s satisfaction at week 48 from baseline, using a 100 mm analogue visual scale.

## 8.2. PROCEDURES FOR THE STUDY PARAMETERS

### 8.2.1. Clinical History and Physical Examination

Demographic and HIV infection-related data will be collected in order to characterize the study population (sex, age, time since HIV diagnosis, risk factor, clinical stage, history of opportunistic infections or tumours and previous antiretroviral treatment).

A complete physical examination will be performed at the baseline visit, including weight and height. In the follow-up, a physical exam will be performed.

In baseline and W48, in order to calculate the cardiovascular risk using the SCORE equation, smoking habits will be recovered and blood pressure will be measured.

### 8.2.2. Laboratory Test

Patients will fast for at least 8 hours prior to assessment, in the points specified in the flow chart of the study (section 8.3). The following parameters will be quantified, as needed:

- **Haematology:**

Leucocyte Hemoglobin

Lymphocyte count Hematocrit

Red blood cell count Platelet count

- **Biochemistry:**

Glucose Alanine aminotransferase (ALT)

Urea Gamma‑GT (GGT)

Creatinine Alkaline phosphatase

SodiumTotal Cholesterol

Potassium HDL Cholesterol

Total Bilirubin LDL Cholesterol

Total Protein Triglycerides

Aspartate aminotransferase (AST)

- **Immunology**

CD4/CD8 Index

CD4 / CD8 percentage

CD4 / CD8 count

- **Microbiology**

Viral load

- **Pregnancy test in urine in women**

**Note: Before the beginning of the study, all labs will facilitate to the sponsor and to the investigator a list of the reference normal values of the parameters to evaluate during the study.**

## 8.3. TRIAL DEVELOPMENT

After accepting the participation, patients would be assessed the first visit. Study visits would be at entry (BL) and weeks 4, 12, 24, 36 and 48.

Plasma samples will be stored at –80ºC.

Viral load, complete blood count, CD4 cells count, and a complete biochemistry and haematology will be performed at every visit (except for week 4 where only CD4 count and viral load will be performed).

**Flow chart of study procedures:**

|  | SCR | BL | Wk 4 | Wk 12 | Wk 24 | Wk 36 | Wk 48 |
| --- | --- | --- | --- | --- | --- | --- | --- |
| Informed consent |  | √ |  |  |  |  |  |
| Pregnancy test |  | √ |  |  |  |  |  |
| Clinical visit |  | √ | √ | √ | √ | √ | √ |
| HIV1-1 Viral load | √ ^a^ | √ | √ | √ | √ | √ | √ |
| CD4 cell count | √ ^a^ | √ | √ | √ | √ | √ | √ |
| Biochemistry (including lipid profile) | √ ^a^ | √ |  | √ | √ | √ | √ |
| Haematology | √ ^a^ | √ |  | √ | √ | √ | √ |
| Patient’s satisfaction |  | √ |  |  | √ |  | √ |
| SCORE equation (blood pressure) |  | √ |  |  |  |  | √ |
| Adverse events |  | √ | √ | √ | √ | √ | √ |
| Plasma sample storage^b^ |  | √ | √ | √ | √ | √ | √ |

^a^ A blood test including HIV-1 RNA, CD4+ counts, biochemistry and haematology performed within 3 months previous to the baseline visit will be accepted as screening.

^b^ Genotypic resistance testing will be performed to all participants experiencing virologic failure during the study.

# 9.- ADVERSE EVENTS

## 9.1. DEFINITION

**Adverse event**: (AE) An adverse event is any medical event presented by a patient or clinical research subject administered a pharmaceutical product, and which does not necessarily have a causal relation to the treatment.

**Serious adverse event**: (SAE) A serious adverse event is a medical event classified as such and which, regardless of the dose involved:

- causes patient death,
- produces a life-threatening situation for the patient,
- requires or prolongs hospital admission,
- produces important or persistent incapacitation/handicap, or constitutes a congenital defect or anomaly,
- needs action to prevent any of above situations.

Examples of such events are intensive care in an Emergency Service or in the home in a patient with allergic bronchospasm; blood dyscrasias or seizures not giving rise to hospital admission, or the development of drug dependency or abuse.

**Unexpected adverse event**: (UAE) An adverse event related to the product in investigation the nature or intensity of which does not coincide with the information available on the product administered (Investigator manual in the case of a non-authorized research drug).

## 9.2. DESCRIPTION OF THE IMPUTABILITY CRITERIA

The causal relation will be established according to the algorithm of the Spanish Pharmacovigilance System, which contemplates the following categories:

**Definitive:**

- A plausible time sequence exists in relation to administration of the drug or its plasma or tissue concentrations.
- The observed manifestation coincides with the known adverse reactions profile of the implicated drug.
- The event cannot be explained by the concurrent disease or by other drugs or chemical substances.
- Response to withdrawal must be clinically plausible, i.e., the condition improves on discontinuing administration of the drug.
- A positive response to repeat exposure is observed.

**Probable:**

- A reasonable time sequence exists in relation to administration of the drug.
- The observed manifestation coincides with the known adverse reactions profile of the implicated drug.
- The event is unlikely to be explained by the concurrent disease or by other drugs or chemical substances.
- Response to withdrawal is clinically plausible, i.e., the condition improves on discontinuing administration of the drug.
- No repeat exposure is required to complete this definition.

**Possible:**

- A reasonable time sequence exists in relation to administration of the drug.
- The observed manifestation coincides with the known adverse reactions profile of the implicated drug.
- The event might be attributable to the clinical condition of the patient or to other concomitantly administered drugs or chemical substances.
- Information concerning drug withdrawal may be unavailable or confusing.

**Improbable:**

- A clinical event, including anomalous laboratory test findings, with a time relation to administration of the drug which makes a causal association unlikely, and where other drugs, chemical substances or intercurrent disease afford plausible explanations for the observed event.

**Unrelated:**

- None of the above criteria are met.

## 9.3. PROCEDURE FOR REPORTING ADVERSE EVENTS

**The investigator:**

The investigator will immediately notify the study sponsor of any serious and/or unexpected adverse events (see section 9.1.).

The report will be realized during the first 24 hours since the start of the serious adverse event. Notification will be made by means of the adverse events reporting form contained in Appendix V of this protocol. This form will be sent to the sponsor by fax.

Adverse events identified in the course of a clinical trial are to be documented in the database (see Appendix I for CRD content).

All adverse events will be notified in table form in the final report of the clinical trial.

All adverse events will be recorded, regardless of the imputability (i.e., causal) relationship involved, in the corresponding adverse events description form. The latter is found in the CRF of each participant in the study (see Annex I).

Depending on the nature of the condition, each adverse event is to be classified as:

- serious / not serious
- unexpected / expected

The characteristics of the event, its seriousness and "expectability", determine when and how it is to be reported to the health authorities.

The recording of adverse events is the responsibility of the trial investigator team, which should indicate the time of appearance of the event (expressed in the shortest time unit possible), its serious / not serious status, and whether it was expected or unexpected. The intensity of the event (mild, moderate or severe) is to be specified, along with the measures adopted (none, treatment, exclusion), course (complete remission, partial remission, persistence) and imputability based on the criteria indicated in section 9.2.

Intensity will be assessed by the investigator using the following terms according to the OMS criteria:

- Grade I-II
- Grade III-IV

**The sponsor:**

The sponsor will inform the Spanish Drug Agency (Ministry of Health), the competent authorities of the autonomous region and the Ethics Committees implicated in the clinical trial about any important information of security of the investigational medicinal product.

The sponsor will inform the Spanish Drug Agency (Ministry of Health) of any serious and unexpected adverse events which may be related to the study treatment, when the investigational medicinal product is not marketed. When it is marketed, it will be informed by means of requirements of pharmacovigilance.

The sponsor will inform competent authorities of the implicated autonomous region and the Ethics Committee of any serious and unexpected adverse events which may be related to the study treatment, and that have been happened in patients in its autonomous region.

The deadlines to notify suspect adverse reaction are:

- 15 days
- 7 days if the suspect adverse reaction has resolved in death or has been life-threatening. The information will be completed in 8 further days.

If the notification is sent in electronic form, it is not necessary to notify the competent authorities of the autonomous region.

The sponsor will keep a detailed register of all the adverse events notified by the investigators.

# 10.- ETHICAL ASPECTS

## 10.1. GENERAL CONSIDERATIONS

### 10.1.1. Declaration of Helsinki

The trial will be conduction in compliance with the principles of the last version of the Declaration of Helsinki

### 10.1.2. Ethics Committees and Regulatory Authority

The present study will be carried out in accordance with Spanish legislation, and the documentation required prior to initiation of the trial is the following:

- Protocol acceptance by the sponsor and the principal investigator
- Protocol approval by the Ethics Committee.
- Protocol authorization from the Spanish Drug Agency (Ministry of Health)

All subjects will be guaranteed continued medical and nursing supervision throughout the duration of the study.

## 10.2. PATIENT INFORMATION AND INFORMED CONSENT

Informed consent will be obtained before including the patient in the trial (Appendix III). The investigator is to inform the patient of the nature, duration and purpose of the study, as well as of all the obstacles and inconveniences which – within reason – may be expected from it. Furthermore, the patient is to receive information in writing. The participating patients must be legally competent to give informed consent, with the possibility of taking decisions at his/her own free will. The patient has the right to leave the study at any time.

## 10.3. CONFIDENTIALITY AND PATIENT PROTECTION

The processing of the data to be compiled by the study sponsor during the trial will be subject to current legislation as regards data protection. The patient will be identified in the records by the corresponding code number only. The patient is to be guaranteed anonymity, and is to be informed that all communication will take place between him/her and the investigator – not the sponsor of the trial.

# 11.- PRACTICAL ASPECTS

## 11.1. RESPONSABILITIES OF STUDY PARTICIPANTS

Obligations of the investigator

The investigators have to conduct the trial in accordance with the established design, and following all the indications of the present protocol or subsequent amendments.

Auxiliary staff

The auxiliary staff collaborating in the study has to follow the established norms and instructions of the investigators. They are also required to report any possible incidents to the investigators.

Norms for patients

The patients will be duly informed and instructed to adhere to the recommendations which make the study possible, as contemplated in this protocol.

## 11.2. PROTOCOL AMENDMENTS

Any changes, amendments or additions to the protocol will be signed by the sponsor and the principal investigator, and must be reported to the Clinical Research Ethics Committee and the Ministry of Health.

Any relevant protocol amendment will require a prior opinion from the Clinical Research Ethics Committee, and authorization from the Spanish Drug Agency.

## 11.3. DATA COLLECTION AND FILING

Data collection will be done directly in an electronic format. This will be carried out by the investigator and his/her collaborators, who will complete the database in accordance with the standard operative procedures (SOPs), in observation of the study protocol.

The information reflected in the database will be regarded as the primary data, with the exception of the patient filiations. The clinical history should also reflect patient participation in the trial, as well as the assigned code number and identification of the different visits taking place during the course of the study.

The data file will adapt to the SOPs of the sponsor procedures.

The subject identification list and written informed consent sheets will be filed separately in the file of the investigator, which will be filed in the same way in the corresponding site.

## 11.4. GOOD CLINICAL PRACTICE (GCP)

This clinical trial will comply with Good Clinical Practice (GCP).

The sponsor and sanitary authorities can audit and inspect the clinical study.

## 11.5 PUBLICATION

Publication of the results of the clinical trial will adhere to the specifications contained in article 38 of Royal Decree 223/2004.

## 11.6. STUDY DOCUMENTATION

1. Protocol.
2. APPENDIX I: CASE REPORT FORM (CRF) CONTENT
3. APPENDIX II: INVESTIGATOR’S BROCHURE
4. APPENDIX III: PATIENT INFORMATION AND WRITTEN INFORMED CONSENT
5. APPENDIX IV: INSURANCE
6. APPENDIX V: NOTIFICATION FORM OF SERIOUS ADVERSE EVENTS
7. APPENDIX VI: REFERENCES

# 12.- STATISTICAL ANALYSIS

The statistical analysis and data management is going to be realized by Lluita contra la SIDA Foundation. An informatized data base of the study is going to be created, it is going to be used to data management too. The statistical analysis is going to be carried out by the statistical programs *SPSS 15 and S-Plus.*

## 12.1. SAMPLE DESCRIPTION

A general descriptive analysis of all the variables of the study, overall and separately by groups of treatment is going to be done using mean, standard deviation, median, interquartile range, maximum and minimum values for the quantitative variables and absolute and relative frequencies of each category for categorical variables.

## 12.2. RESPONSE EVALUATION

The response evaluation is going to be done per-protocol, an interim analysis will be performed will be performed when 75% of subjects reach week 24 of follow-up , the final analysis will be based on patients completing the entire 48 weeks of treatment.

The statistical significance of the longitudinal changes in the HIV RNA viral load and lipid parameters are going to be assessed by the calculation of the slope of decay and compared using t-student, Wilcoxon or Mann-Whitney distinguishing between treatment groups and/or other relevant baseline factor.

HIV RNA viral load is going to be transformed by the log_10_ and if necessary, other transformations of the data are going to be considered.

## 12.3. SAFETY ANALYSIS

The information about drop out and tolerability to the treatment (physical examination and clinical laboratory tests) is going to be analyzed in a graphical and/or tabular descriptive form for all the patients included in the study. The clinical relevance of the values of each control as well as the changes happened between different controls is going to be evaluated.

All the adverse events are going to be tabulated and analyzed descriptively.

## 12.4. SAMPLE SIZE DESCRIPTION

This is a control-armed pilot study designed to examine the proof-of-concept of switching from a PI to etravirine.

Given the lack of data with this strategy an exploratory close monitored pilot trial with a limited sample size would provide useful information for the confirmation of the hypothesis and the design of future, adequately powered trials.

The study is going to be carried out in a sample of 40 patients (20 patients in experimental group and 20 patients in control group).

# Appendix I: CASE REPORT FORM (CRF)

**(Attached)**

# Appendix II: INVESTIGATOR’S BROCHURE

**(Attached)**

# Appendix III: PATIENT INFORMATION AND WRITTEN INFORMED CONSENT

**(Attached)**

# Appendix IV: INSURANCE

**(Attached)**

Appendix V: NOTIFICATION FORM OF SERIOUS ADVERSE EVENTS

**(Attached)**

# Appendix VI: REFERENCES

1. Andries K, Azijn H, Thielemans T, et al. TMC125, a novel next-generation nonnucleoside reverse transcriptase inhibitor active against nonnucleoside reverse transcriptase inhibitor-resistant human immunodeficiency virus type 1. *Antimicrob Agents Chemother*, 2004. 48(12):4680-6
2. Panel on Antiretroviral Guidelines for Adults and Adolescents. Guidelines for the use of antiretroviral agents in HIV-1-infected adults and adolescents. Department of Health and Human Services. November 3, 2008; 1-139. Available at http://www.aidsinfo.nih.gov/ContentFiles/AdultandAdolescentGL.pdf. (Accessed Nov 26th, 2008).
3. Ruxrungtham K, Pedro RJ, Latiff GH, et al. Impact of reverse transcriptase resistance on the efficacy of TMC125 (etravirine) with two nucleoside reverse transcriptase inhibitors in protease inhibitor-naïve, nonnucleoside reverse transcriptase inhibitor-experienced patients: study TMC125-C227. *HIV Med*, 2008. 9(10):883-96.
4. Haubrich R, Cahn P, Grinsztejn B, et al; DUET-1 study group. DUET-1: Week 48 results of a Phase III randomized double-blind trial to evaluate the efficacy and safety of TMC125 vs placebo in 612 treatment-experienced HIV-1-infected patients. In: Program and abstracts of the 15th Conference on Retroviruses and Opportunistic Infections; February 3-6, 2008; Boston. Abstract 790.
5. Johnson M, Campbell T, Clotet B, et al; DUET-2 study group. DUET-2: Week 48 results of a Phase III randomized double-blind trial to evaluate the efficacy and safety of TMC125 vs placebo in 591 treatment-experienced HIV-1-infected patients. In: Program and abstracts of the 15th Conference on Retroviruses and Opportunistic Infections; February 3-6, 2008; Boston. Abstract 791.
6. Lazzarin A, Campbell T, Clotet B, et al. Efficacy and safety of TMC125 (etravirine) in treatment-experienced HIV-1-infected patients in DUET-2: 24-week results from a randomised, double-blind, placebo-controlled trial. *Lancet*, 2007. 370(9581):39-48 .
7. Madruga JV, Cahn P, Grinsztejn B, et al. Efficacy and safety of TMC125 (etravirine) in treatment-experienced HIV-1-infected patients in DUET-1: 24-week results from a randomised, double-blind, placebo-controlled trial. *Lancet*, 2007. 370(9581):29-38
8. Woodfall B, Vingerhoets J, Peeters M, et al. Impact of NNRTI and NRTI resistance on the response to the regimen of TMC125 plus two NRTIs in Study TMC125-C227. In: Program and abstracts of the 8th International Congress on Drug Therapy in HIV Infection; November 12-16, 2006; Glasgow, United Kingdom. Abstract PL5.6.
